# Spatial Atomic Layer Deposition of IrO_*x*_ Using (EtCp)Ir(CHD) and Atmospheric O_2_/N_2_ Plasma

**DOI:** 10.1021/acs.jpcc.5c06502

**Published:** 2025-11-27

**Authors:** Mike L. van de Poll, Jie Shen, Paul Poodt, Fieke van den Bruele, Wilhelmus M. M. Kessels, Bart Macco

**Affiliations:** † Department of Applied Physics and Science Education, 3169 Eindhoven University of Technology, 5600 MB Eindhoven, The Netherlands; ‡ 338498 TNO/Holst Centre, High Tech Campus 31, 5656 AE Eindhoven, The Netherlands; § SparkNano B.V., Esp 266, 5633 AC Eindhoven, The Netherlands

## Abstract

High-volume production of stable and affordable electrocatalysts is essential for the large-scale green hydrogen production using proton exchange membrane (PEM) water electrolysis. Iridium oxide (IrO_*x*_) is a leading catalyst for the oxygen evolution reaction (OER) due to its high activity and stability in acidic conditions, but its cost and scarcity require strategies to minimize Ir usage. One promising approach is the deposition of ultrathin IrO_*x*_ films on porous substrates using spatial atomic layer deposition (SALD), which offers precise thickness control, excellent conformality, combined with high throughput. In this work, a SALD IrO_*x*_ process using (EtCp)­Ir­(CHD) and atmospheric O_2_/N_2_ plasma was developed. The process exhibits saturated growth with a growth-per-cycle of 0.66 Å at 150 °C. The impurity content in the films decreases sharply with increasing temperature between 80–150 °C, while metallic Ir clusters begin to form above ∼150–180 °C. Extended plasma exposure beyond saturation further reduces impurities and leads to denser, more crystalline films. Post-deposition anneal (PDA) in O_2_ atmosphere was shown to fully convert the deposited films into stoichiometric crystalline IrO_2_. Moreover, the conformality was studied on lateral high-aspect-ratio test structures and shown to be sufficient for depositions inside porous transport layers in PEM cells. Finally, the oxygen radical recombination probability on IrO_*x*_ during the deposition was determined to be in the order of 10^–3^.

## Graphical abstract

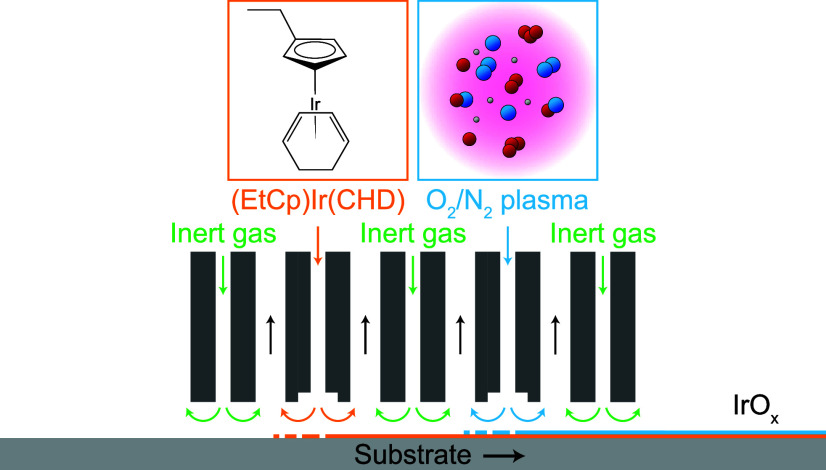

## Introduction

To realize the green energy transition, proton exchange membrane (PEM) water electrolyzers have attracted significant interest for green hydrogen production.^1–3^ A clear benefit of PEM over other methods like alkaline water electrolysis (AWE) is that PEM systems respond much more quickly to changes in power input, making them ideal for pairing with intermittent renewable energy sources like solar and wind.^4^ While they also offer high current densities, a high voltage efficiency, and a high gas purity, their expensive components pose a challenge for large-scale adoption.^1^ A significant part of the component costs come from the noble metals that are used as electrocatalyst on the anode for the oxygen evolution reaction (OER). The use of these metals is required for their electrocatalytic activity and stability in the harsh acidic conditions of the PEM electrolysis cell. Ir-based materials are of particular interest since they exhibit the best balance between activity and stability.^5–7^

Since Ir is extremely scarce and expensive, reduction of the catalyst loading is paramount for commercialization. State-of-the-art PEM electrolysis cells contain Ir-loadings of 1–2 mg cm^–2^ in the form of a catalyst coated membrane (CCM).^3,8^ The CCM is brought in contact with a metallic porous transport layer (PTL), which electrically contacts the catalyst and facilitates mass transport of H_2_O and O_2_. This design has several downsides, one of which is the low active surface area of the thick (several μm) CCM relative to the Ir-loading.^1^ The thick layer also imposes a long ionic diffusion length on the generated protons to reach the other side of the membrane. Another issue is that local contact of the CCM with the PTL can lead to localized hot spots due to uneven current distribution, which can damage the catalyst layer and membrane.^9^

Many different Ir-based materials have been shown to exhibit high OER activity, and to determine the optimal material, the catalytic activity and stability of these materials need to be considered. Generally, the OER activity and stability of different materials show an inverse relation and it is therefore essential to reach a balance between the two.^5,7,10,11^ The specific OER activities of various Ir-based materials show the following trend: perovskites ≈ metallic Ir ≈ amorphous IrO_*x*_ ≫ crystalline IrO_2_.^12^ The difference in specific activity is caused by the formation of a highly active amorphous IrO_*x*_ layer on the SrIrO_3_ and metallic Ir, which does not form on the stable crystalline IrO_2_. This IrO_*x*_ layer is however prone to dissolution. To quantify the balance between the activity and stability, the S-number is used, which is defined as the ratio between the amount of evolved O_2_ and dissolved Ir.^12^ Because of the stability of crystalline IrO_2_, its S-number is 2–3 orders of magnitude higher than the other aforementioned Ir-based materials. Therefore, development of ultrathin-polycrystalline-IrO_2_-film production methods, where the high surface area compensates for the lower intrinsic activity, is highly promising for improving PEM water electrolyzers.

An appealing production method for the thin IrO_*x*_ catalyst layer is atomic layer deposition (ALD).^13^ ALD is a vapor-phase deposition technique consisting of alternating half-cycles with self-limiting surface reactions.^14^ In the first half-cycle the substrate is exposed to a metal precursor, which reacts with the surface, after which any remaining precursor and reaction products are purged away. In the second half-cycle the substrate is exposed to a co-reactant, which reacts with the surface, followed by another purge step. Since the chemical reactions in ALD are self-limiting surface reactions, repeating these cycles results in uniform, virtually pinhole free films with excellent thickness control. These characteristics make the technique promising for the deposition of a-few-nm-thick IrO_*x*_ films for the OER, leading to reduced Ir-loading for the same active surface area. Additionally, ALD IrO_*x*_ can be directly deposited on the PTL, thus increasing the surface area of the catalyst layer (Figure 1). The resulting so-called porous transport electrode (PTE) has been shown to outperform CCM at high current densities,^15^ and has the added benefit of being better suited for continuous production.^13^

**Figure 1:**
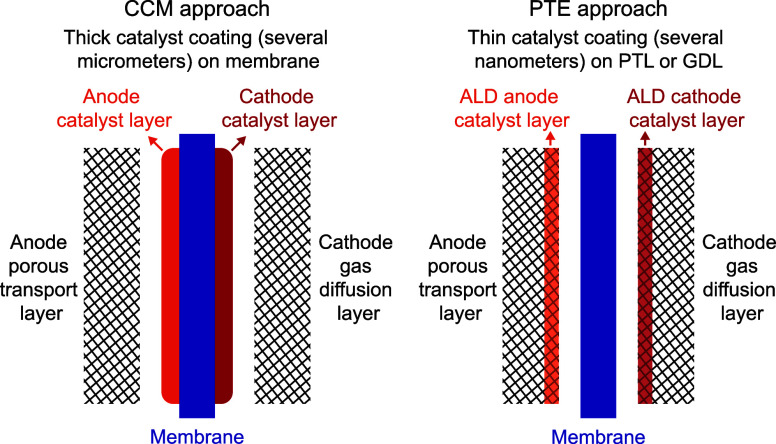
Schematic comparison of the catalyst coated membrane (CCM) and porous transport electrode (PTE) approaches. In CCM, the catalyst layers are applied on the membrane, while in PTE they are applied on the porous transport layer (PTE) for the anode, and on the gas diffusion layer (GDL) for the cathode.

Spatial ALD (SALD) is a variant of ALD that is particularly interesting for high-throughput production of PTEs.^16^ In SALD, the precursor and co-reactant vapors are separated spatially rather than temporally, by dosing them simultaneously in distinct zones separated by a flow of inert gas. ALD cycles are created by moving the substrate through the different reactant zones. SALD is ideal for high-volume applications, since it is significantly faster than temporal ALD, easier to scale up to large substrate sizes, and it can be performed at atmospheric pressure. Furthermore, Niazi et al. have discussed that SALD has a lower environmental footprint than temporal ALD, which is mainly attributed to its lower energy consumption.^17^ Moreover, it is compatible with different continuous processing modes such as roll-to-roll and sheet-to-sheet.^18,19^

Several IrO_*x*_ processes have been reported so far.^20^ The used Ir-precursors always have organic ligands, and are typically either Ir­(acac)_3_,^21–25^ or a molecule containing two carbon rings (i.e., (MeCp)­Ir­(CHD),^26^ (EtCp)­Ir­(CHD),^27–29^ (MeCp)­Ir­(COD),^30^ (EtCp)­Ir­(COD),^31^ where Cp = cyclopentadienyl, CHD = cyclohexadiene, and COD = cyclooctadiene). Since the Gibbs free energy of formation Δ*G*_IrO_2__ has a relatively small negative value, strong oxidizing agents are required as co-reactant, such as O_3_ and O_2_ plasma, although Kim et al. have shown that high partial pressures of O_2_ gas also work at specific conditions.^31^ If less strong oxidizing agents are used or the deposition temperature is too high, metallic Ir is deposited instead. The observed upper temperature limit is typically between 150 and 200 °C.^21,28,32^ In this sense, these ALD processes are very similar to those of oxides of other platinum-group metals, such as Ru, Pd, and Pt.^33–35^ ALD IrO_*x*_ films are typically nanocrystalline with rutile crystal structure. The crystallinity of the films increases with increasing deposition temperatures, as is a general trend for ALD of crystalline materials.^26,36^ The lowest deposition temperature resulting in crystalline films is around 120 °C.^28^ Despite the large interest in thin IrO_*x*_ films, no SALD IrO_*x*_ processes have been reported in the literature yet, while immense upscaling is required for high-volume manufacturing of PEM electrolyzers.

In this work, an atmospheric-pressure SALD IrO_*x*_ process using (EtCp)­Ir­(CHD) and atmospheric O_2_/N_2_ plasma was developed. The influence of the deposition temperature and plasma exposure time on the film properties was investigated, where higher deposition temperatures and extended plasma exposures result in more crystalline films with a lower impurity content. Moreover, post-deposition anneal (PDA) strategies were explored for improving the film properties. Especially PDA in O_2_ atmosphere proved interesting, as it resulted in crystalline IrO_2_. Furthermore, the growth on application-relevant Ti substrates was studied, showing no substantial differences to the process developed on silicon wafers. Finally, the conformality of the process was characterized by performing depositions inside lateral high-aspect-ratio (LHAR) trench structures.^37^

## Experimental Methods

### IrO_*x*_ Thin Film Preparation

IrO_*x*_ thin films were deposited using a home-built atmospheric-pressure SALD reactor with rotating substrate table. The reactor is described in more detail by Poodt et al.^38^ Ethylcyclopentadienyl cyclo-hexadiene iridium­(I) ((EtCp)­Ir­(CHD)) was used as precursor. The precursor was supplied through Ar bubbling from a stainless steel canister held at 80 °C. Precursor exposures were calculated assuming a vapor pressure of 0.12 Torr at 80 °C, based on the reported vapor pressure of 0.1 Torr at 75 °C by Kawano et al.^39^ The co-reactant slot of the deposition head was equipped with a home-built close-proximity remote dielectric-barrier-discharge (DBD) plasma source, described by Creyghton et al.^40^ An O_2_/N_2_ plasma (100 sccm O_2_ and 9900 sccm N_2_) was used as co-reactant. N_2_ gas was chosen over Ar gas because of cost considerations, while the low the low O_2_ fraction was specifically selected to suppress the conversion of O radicals to O_3_ (i.e., O + O_2_ → O_3_). The exposure times are defined as the slot widths divided by the substrate speed. In practice, the precursor and radical concentration in the slots will likely vary along the slot length, especially in the plasma slot, where the radical density will be confined to the center of the slot. Hence, the exposure time used here is purely for comparing results within this work and not with other (Spatial) ALD reactors and plasma sources. A more detailed description of exposure and exposure time calculations can be found in the Supporting Information.

The majority of the films were deposited on Si wafers. Additional depositions were performed on Ti substrates to mimic the substrate material used in PTEs. The conformality of the process was studied by depositing on PillarHall test chips (Chipmetrics Ltd.).^37^ These Si chips contain lateral high-aspect-ratio (LHAR) trench structures of which the top membrane can easily be removed to investigate the film that has penetrated into the structures.

Select samples underwent post-deposition annealing (PDA) in either 1 atm of pure O_2_, N_2_, or an N_2_/H_2_ mixture using a Jipelec rapid thermal anneal (RTA) furnace. The temperature was ramped up to either 300 or 500 °C in 10 s, and held for 5 min. Subsequently, the temperature decreased back to room temperature without the use of active cooling.

### Film Characterization

Spectroscopic ellipsometry (SE) measurements were performed with a J. A. Woollam Co. Inc. M-2000 spectrometer. Thickness and refractive index were determined by fitting Ψ and Δ over a spectral range of 1.2 to 4.8 eV using three Lorentz oscillators and a Drude oscillator. The Lorentz peak positions were limited to specific energy ranges (i.e., 0.9–1.6, 3.5–4.0, and >7.0 eV) during fitting of the model, based on the work by Sachse et al.^41^ An example of the resulting dielectric functions can be seen in Figure S1. X-ray reflectivity (XRR) and grazing-incidence X-ray diffraction (GI-XRD) measurements were performed using a Bruker D8 DISCOVER system with Cu Kα (λ = 1.54060 Å) radiation. XRR was used to determine the thickness and mass density of the films, GI-XRD was used to study their crystallinity. X-ray fluorescence (XRF) measurements were performed using a Fischerscope X-ray XDV-SDD spectrometer to measure the Ir-loading and determine the density of Ir-atoms. The chemical composition of the films was analyzed by performing X-ray photoelectron spectroscopy (XPS) measurements using a Thermo Scientific K-Alpha XPS system with monochromated Al Kα source (λ = 8.3386 Å). The lateral resistivity of the films at room temperature was determined using a Signatone four-point probe and a Keithley 2400 source meter. The surface morphology was studied by performing atomic force microscopy (AFM) measurements using a Bruker Dimension Icon. Finally, optical microscopy images were taken of the films deposited inside LHAR structures using an Olympus BX53 to study the conformality of the process. Gray-scale analysis was performed on the images by Chipmetrics Ltd. to obtain relative-thickness profiles, which were combined with SE measurements on planar substrates to obtain absolute-thickness profiles.

## Results and Discussion

### Saturation of the ALD Process

The self-limiting behavior of the ALD process was studied by changing the rotation frequency of the substrate table, which influences the precursor exposure and purge times, and plasma exposure and purge times simultaneously. The resulting growth per cycle (GPC) as a function of precursor and plasma exposure for a deposition temperature of 150 °C and precursor flow of 150 sccm is shown in Figure 2. The growth saturates at 0.66 Å for ∼290 ms of precursor exposure time—corresponding to an exposure of 5.2 × 10^3^ L—and ∼790 ms of plasma exposure. The GPC matches the GPC of 0.66 Å reported by Simon et al., however they used a slightly different precursor, i.e., (MeCp)­Ir­(COD) instead of (EtCp)­Ir­(CHD), and a deposition temperature of 110 °C instead of 150 °C.^30^ The only other (EtCp)­Ir­(CHD)/O_2_ plasma process reported in literature by Di Palma et al. has a significantly lower GPC of 0.28 Å at 150 °C.^29^ The process exhibits linear growth and a nucleation delay of around 50 cycles (Figure S2), aligning with the nucleation delay reported for temporal ALD.^29^ Whether the precursor or plasma dose is the limiting factor for reaching saturated growth cannot be determined from the data shown in Figure 2, and will be investigated further below.

**Figure 2:**
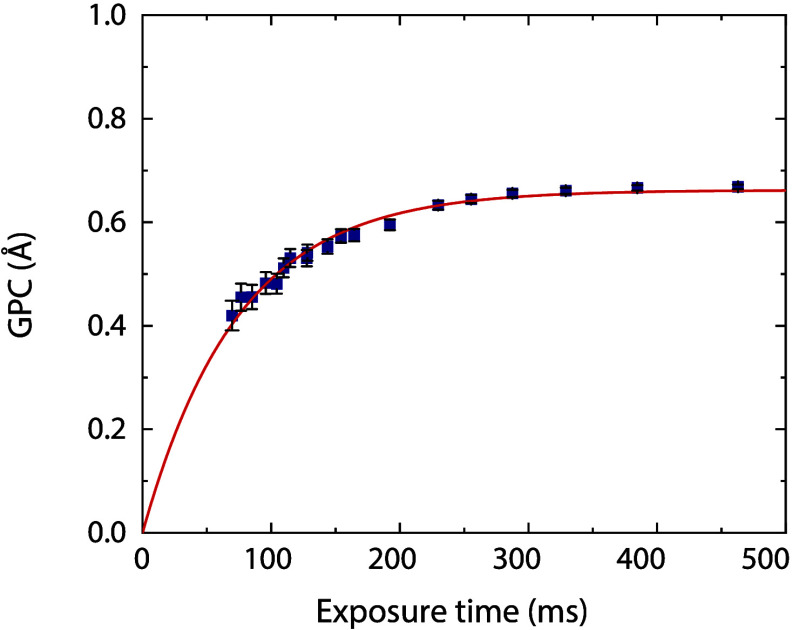
GPC of SALD IrO_*x*_ at 150 °C as a function of the precursor and plasma exposure times, which were changed simultaneously by varying the rotation frequency of the substrate table.

### Influence of the Deposition Temperature

After validating the self-limiting behavior of the process, the influence of the deposition temperature on the process and film properties was determined, by varying the deposition temperature from 80 to 200 °C (Figure 3). The GPC drops with increasing temperature, from 1.32 Å at 80 °C to 0.40 Å at 200 °C (Figure 3a). While the films deposited at temperatures below 180 °C appeared uniform, those deposited at 180 and 200 °C visually show additional reflective regions, indicative of metallic film. GI-XRD patterns of the films are shown in Figure 3b. The films deposited at 180 °C and below only show broad features around 2θ values of 30 and 42°, and no other reflections, indicating that these films are mostly amorphous. The distinct reflections from rutile IrO_2_ and metallic Ir are not observed.^42,43^ The film deposited at 200 °C shows signal at 2θ values of 41, 47, and 69, corresponding with the (111), (200), and (220) reflections of metallic Ir, respectively. The peak around the 2θ value of 52 comes from the Si substrate and the exact location and intensity of the peak is dependent on the orientation of the sample with respect to the beam.

**Figure 3:**
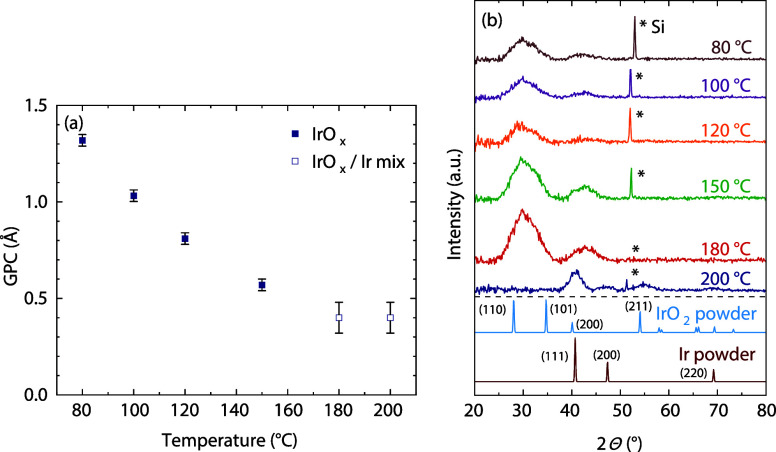
(a) GPC of the PE-SALD IrO_*x*_ process as a function of the deposition temperature as measured by SE and (b) GI-XRD measurement data of the resulting films and IrO_2_ and Ir powder reference data.^42,43^

The deposited films were further analyzed using XPS (Figure 4). The measurements of the 180 and 200 °C samples were specifically performed on metallic-like spots. The Ir 4f peak shifts to lower binding energies as the temperature increases (Figure 4a), indicating a reduced oxidation state of the Ir atoms. Between 80 and 150 °C, the maximum intensity shifts slightly from binding energies associated with IrO_*x*_, with *x* > 2, toward binding energies associated with more stoichiometric IrO_2_.^44,45^ At 180 and especially 200 °C, the peak maximum shifts to binding energies associated with metallic Ir, and the peak becomes narrower and more intense.^44,45^ The O 1s peak follows a similar trend (Figure 4b), where a shoulder appears at 530 eV at increased temperatures, corresponding to IrO_2_. Above 150 °C, the overall intensity of the peak lowers, and a shoulder appears at 533 eV. This position corresponds to SiO_2_, indicating that the metallic Ir film partially dewets from the substrate, exposing the native SiO_2_ layer underneath, similar as shown further below for other metallic Ir films. Analysis of the ratio of Ir atoms to O atoms (Figure 4d) reveals substoichiometric Ir contents (i.e., IrO_*x*_ with *x* > 2) at low temperatures. For increasing temperatures up to 150 °C the ratio slightly increases, while rapidly increases at higher temperatures. The latter trend confirms that increasing parts of the film are metallic, in line with the GI-XRD results. The XPS data reveals that the deposition-temperature threshold for partial metallic Ir films is between 150 and 180 °C, which was not evident from the GI-XRD data alone due to the low amount of metallic Ir present in the 180 °C film. The observed transition between IrO_*x*_ and Ir at a critical deposition temperature in this temperature range is in line with reports in the literature.^21,28,32^

**Figure 4:**
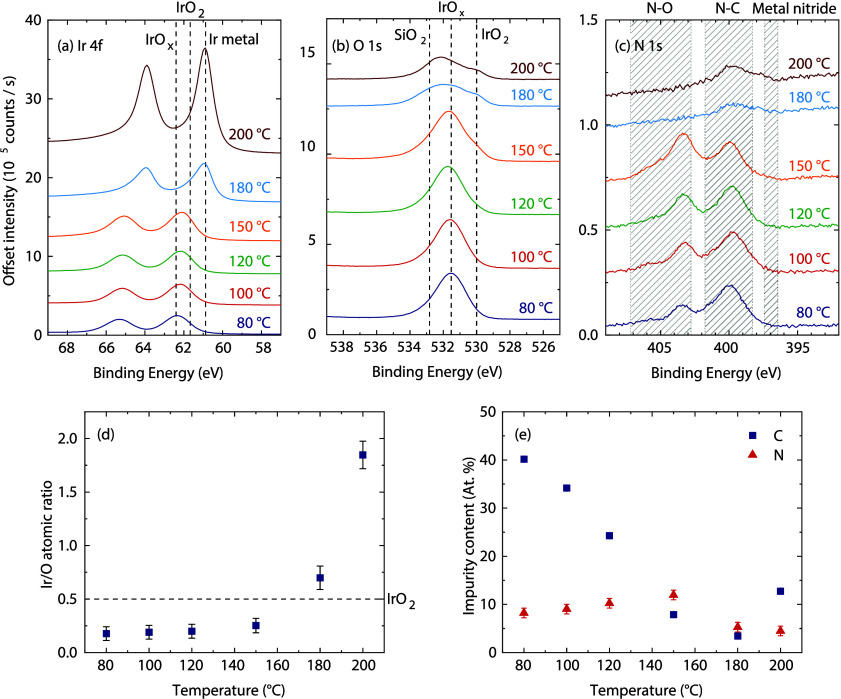
XPS measurements of IrO_*x*_ films deposited at various deposition temperature: (a) Ir 4f spectra, (b) O 1s spectra, (c) N 1s spectra, (d) ratio of Ir atoms to the sum of Ir and O atoms, and (e) impurity content.

XPS analysis also reveals a significant amount of C and N impurities in the films (Figure 4e). The C content drops from 40 to 7.9 atomic % over the temperature range of 80 to 150 °C. Some C content is often observed in ALD films and is typically the result of incomplete removal of organic precursor ligands. Increasing the deposition temperature helps ligand removal by increasing the reactivity between the co-reactant and the ligands, and by promoting ligand desorption from the surface. In the range of 80 to 150 °C, the N content slightly increases from 8.2 to 12 atomic %. The N 1s signal consists of two peaks (Figure 4c) indicating that the N atoms have at least two distinct chemical environments. One of the peaks is positioned around 403.5 eV, which falls within a region corresponding to N–O containing compounds.^46^ This peak disappears above 150 °C, when the O content in the film drops. The other peak has its maximum around 400 eV, falling within a region corresponding to N bound to C.^46^ For metal nitrides, a signal around 397 eV is typically observed, which is not the case here.^47–49^ Moreover, Ir-nitride typically only forms in extreme conditions and has not been reported for ALD or chemical vapor deposition (CVD). Therefore, it can be concluded that the N in these films is primarily bound to O and C, and the films are oxides rather than oxynitrides. Since the precursor is free of N atoms, these impurities most likely originate from reactive N or NO_*x*_ radicals formed in the O_2_/N_2_ plasma,^50,51^ or from NH_*x*_ radicals which might be formed due to trace amounts of water in the plasma. In line with this explanation, Di Palma et al. deposited ALD IrO_*x*_ using the same precursor but in combination with an O_2_/Ar plasma, and observed no significant N content.^29^ Overall, the highest purity IrO_*x*_ without the presence of metallic Ir is obtained at a deposition temperature of 150 °C, which will be used for all subsequent depositions. The results also show that this chemistry can be used successfully for metallic Ir depositions at higher temperatures, but for the investigated range of settings the metallic Ir is mixed with IrO_*x*_. For a study of metallic Ir it would be beneficial to increase the deposition temperature above 200 °C to avoid the formation of a mixed IrO_*x*_/Ir films.

### Influence of the Plasma Exposure Time

The influence of the plasma exposure time on the film growth, composition, and properties was investigated. Plasma exposure is known to play an important role in PE-ALD processes, as it can lead to effects such as impurity removal,^52^ and redeposition.^53^ Ion bombardment is another effect,^54^ but in the present case at atmospheric pressure it is unlikely to be significant because collisions of the ions prevent them from accelerating over the plasma sheet.^55^ Various plasma exposure times were obtained by changing the rotation frequency of the substrate table (Figure 5). The resulting change in precursor exposure time was compensated for by tuning the carrier-gas flow through the precursor bubbler, such that the precursor dose was kept constant.

**Figure 5:**
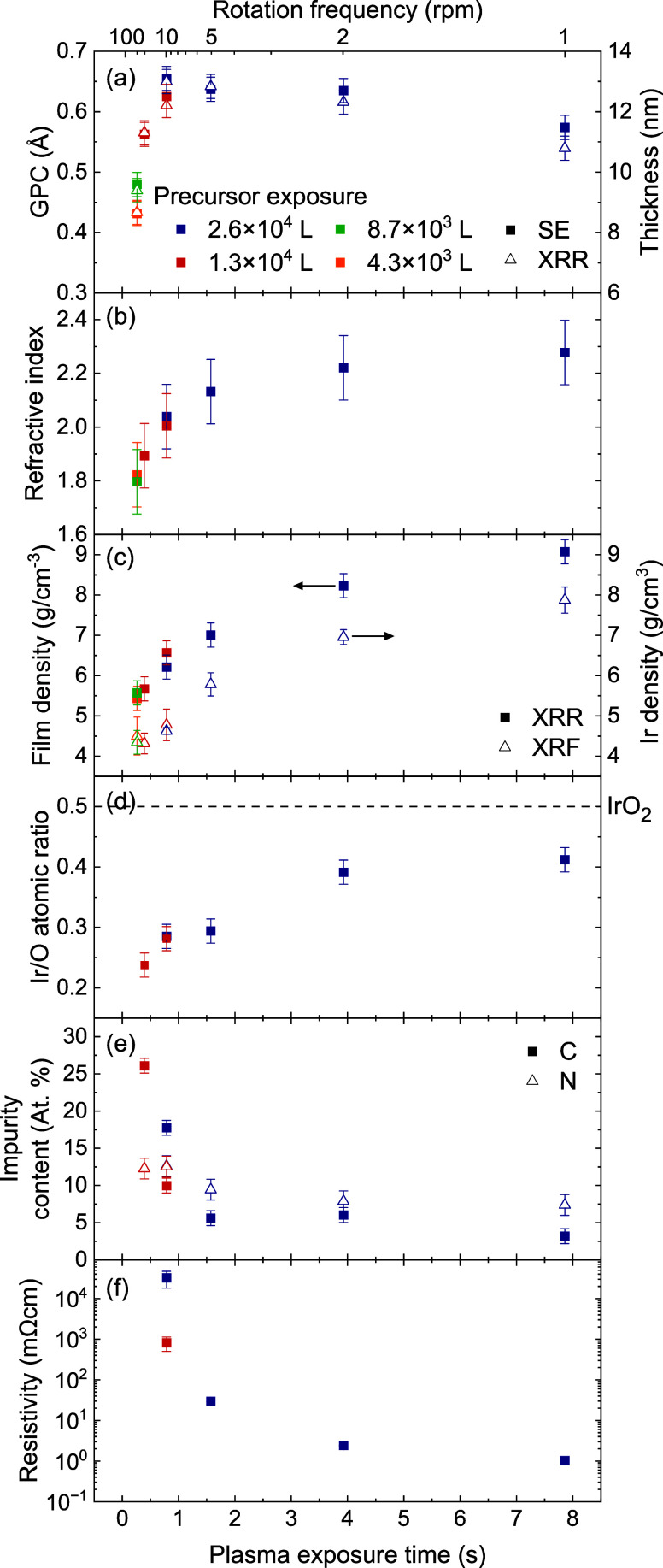
Growth properties, composition, and material properties of PE-SALD IrO_*x*_ films as a function of plasma exposure time. (a) GPC and thickness as determined by spectroscopic ellipsometry (SE) and X-ray reflectivity (XRR). (b) Refractive index as determined by SE. (c) Mass densities of the film and of the Ir atoms as determined by XRR and X-ray fluorescence (XRF), respectively. (d) Ratio of Ir and O atoms as determined by XPS. (e) Atomic percentages of C and N atoms as determined by XPS. (f) Resistivity as determined by four-point probe (FPP) measurements.

Figure 5a shows the GPC as a function of the plasma exposure time. The SE and XRR results show a similar trend, wherein the GPC increases with increasing plasma exposure time up to around 0.66 Å at 0.79 s and subsequently decreases to a value of around 0.5–0.6 Å at 7.9 s. This value of 0.79 s matches the plasma exposure time required for reaching saturated growth as was shown earlier in Figure 2. There, only the rotation frequency was varied, but the results shown in Figure 5a reveal that the plasma exposure was the limiting factor for reaching saturated growth. Figure 5b-e shows that with increased plasma exposure time there is a monotonic and saturating increase in the refractive index, film density and the Ir/O ratio. The film resistivity, and C and N impurity content decrease with increasing plasma exposure time.

The drop in GPC can be explained by a significant reduction in the C impurity content and an increase in the film density. The increased plasma dose likely facilitates further combustion of the precursor ligands, resulting in reduced C incorporation. The density of Ir atoms, as determined by XRF, follows a similar trend to the overall IrO_*x*_ film density, albeit with lower absolute values, as expected. Converting the film density obtained by XRR to Ir atom density using the film composition determined by XPS results in values that are in good agreement with the XRF data (e.g., 5.2 and 5.8 g/cm^–3^ at 1.57 s plasma exposure and 7.3 and 7.9 g/cm^–3^ at 1.57 s plasma exposure). As the plasma exposure time increases, the difference between the film density and Ir density becomes smaller. This is in line with the XPS results, which show that the Ir/O atomic ratio increases with the plasma exposure time toward a more stoichiometric IrO_2_, although all films are Ir-deficient. The Ir 4f and O 1s peaks also shift toward lower binding energies (Figure S3) indicating the increased formation of IrO_2_. In addition to C, the films also contain a considerable amount of N impurities, distributed throughout the bulk of the film. As the plasma exposure is increased the N content decreases from 13 to 7.4 atomic %.

The resistivity of the films (Figure 5f) decreases by several orders of magnitude when extending the plasma exposure. This decrease is likely linked to the reduced impurity content, as well as the improved crystallinity of the films, as will be discussed further below. This explanation is consistent with reports by Lodi et al. and Rasten et al., who observed that the resistivity of amorphous IrO_*x*_ is several orders of magnitude higher than for IrO_*x*_ after crystallization.^56,57^ The lowest resistivity observed is still significantly higher than the room temperature resistivity of bulk rutile IrO_2_ of 49 μΩcm.^58^ This difference likely results from ionized impurity scattering caused by the impurities in the film, and significantly grain boundary scattering caused by small crystal grains.

To determine the influence of the plasma exposure time on the crystallinity, GI-XRD measurements were performed on the films (Figure 6). At a plasma exposure time of 0.79 s, the film is predominantly amorphous. As the plasma exposure time increases, so does the crystallinity, as seen by an increase in intensity of the bands at 2θ = 35 ° and 2θ = 54 ° corresponding to the (101) and (211) facets of rutile IrO_2_.^42^ A likely explanation for this trend is the reduced impurity content in the films for longer plasma exposures. Furthermore, the extended plasma exposure could provide additional energy to the material, thereby promoting nucleation and grain growth. The polycrystalline IrO_*x*_ films have small grains which could not be observed by AFM, meaning that the grain size is likely around 10 nm or smaller. This aligns with the commonly reported trend that the lateral grain size in many polycrystalline ALD films (e.g., ZnO, ZnS, TiN, HfO_2_ films) do not exceed the film thickness.^36^ Furthermore, from the change in band intensities it can be noted that the films are predominantly (211)-textured at short plasma exposure times, and become increasingly (101)-textured at longer plasma exposure times.

**Figure 6:**
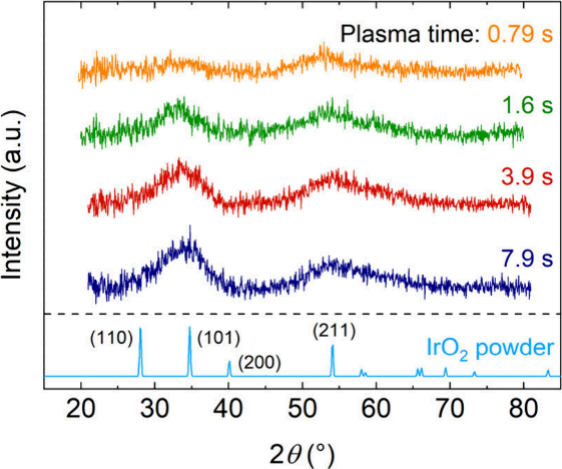
Grazing-incidence X-ray diffraction (GI-XRD) patterns of IrO_*x*_ films deposited at 150 °C using various plasma exposure times, and IrO_2_ powder reference data.^42^ Film thicknesses of these samples decrease slightly with plasma exposure time, from 13.1 nm at 0.79 s to 11.5 nm at 7.9 s.

Overall, the use of an extended plasma exposure is beneficial for increasing the purity, conductivity, and crystallinity of the IrO_*x*_ film. As discussed in the introduction, the latter is essential for creating a stable OER electrocatalyst.

### Post-deposition Anneal

Post-deposition anneal (PDA) of the IrO_*x*_ films was explored as a strategy to further improve their purity and crystallinity. The films used for this experiment were deposited at 150 °C using a rotation frequency of 5 rpm and a precursor carrier-gas flow of 375 sccm, corresponding to a precursor exposure of 2.6 × 10^4^ L and a plasma exposure time of 1.6 s. The thickness of the films before annealing was 12.7 nm. The samples were annealed for 5 min in a rapid thermal anneal (RTA) setup at either 300 or 500 °C, in an N_2_, N_2_/H_2_ (10% H_2_), or O_2_ atmosphere. The crystallinity, chemical composition, and surface morphology were studied using GI-XRD, XPS, and AFM, respectively (Figure 7 and Figure 8).

**Figure 7:**
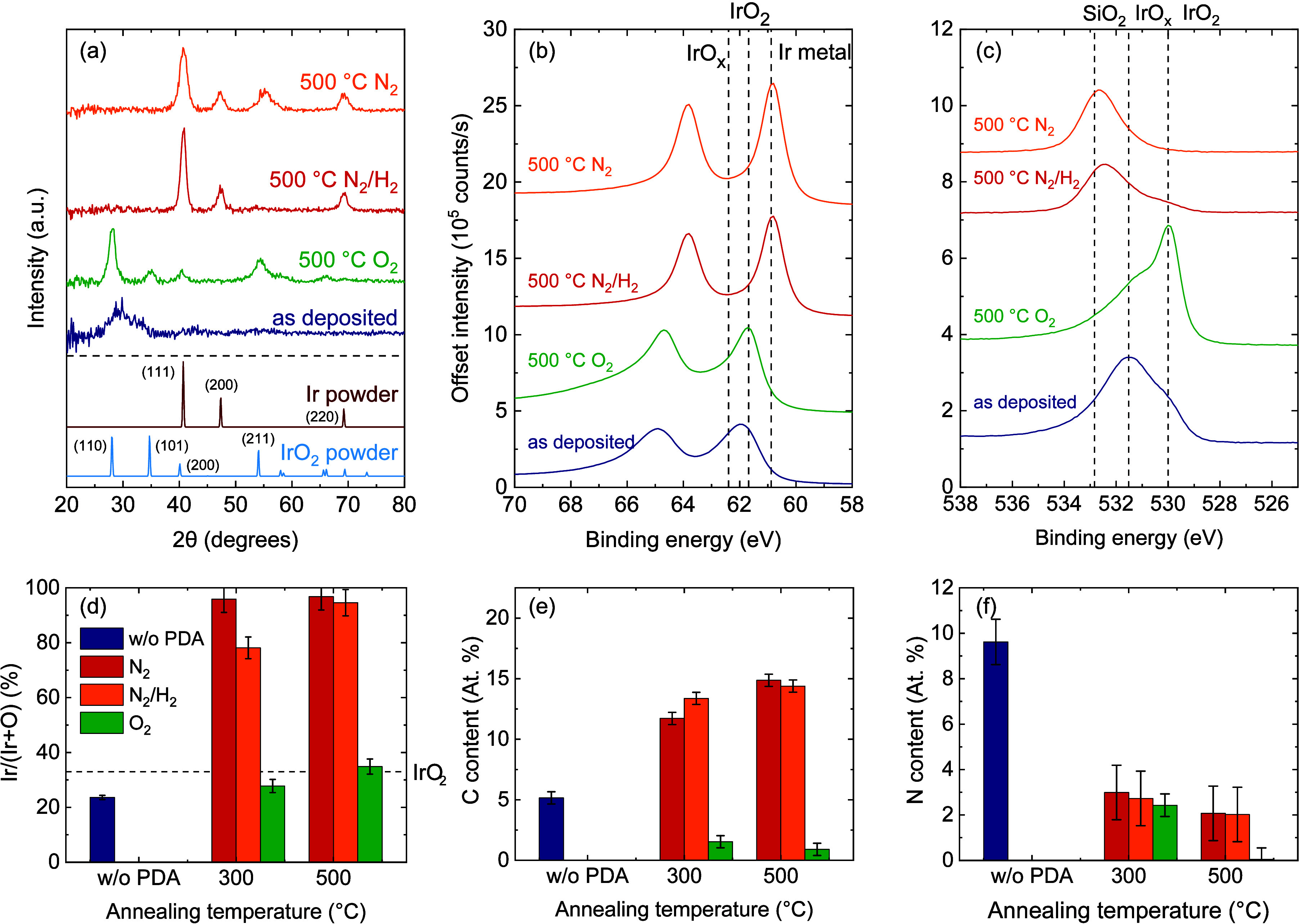
Crystallinity and chemical composition of SALD IrO_*x*_ films with different post-deposition anneal (PDA) treatments. (a) GI-XRD diffractograms and reference patterns,^43,44^ and (b) Ir 4f, and (c) O 1s XPS spectra. (d) Ir/(Ir + O) ratio, (e) C content, and (f) N content of the films as determined by XPS.

**Figure 8:**
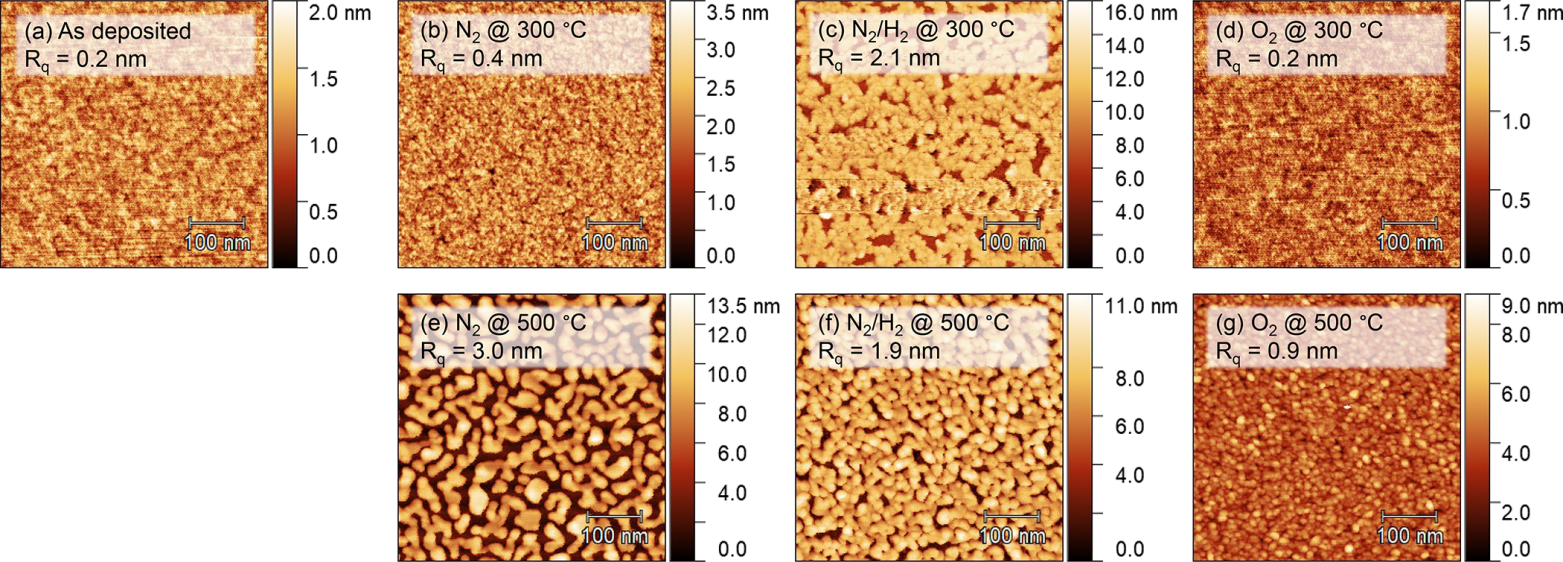
AFM images of SALD IrO_*x*_ films (a) as deposited, and with different post-deposition anneal (PDA) treatments: 5 min in (b) N_2_ at 300 °C, (c) N_2_/H_2_ at 300 °C, (d) O_2_ at 300 °C, (e) N_2_ at 500 °C, (f) N_2_/H_2_ at 500 °C, and (g) O_2_ at 500 °C.

PDA in N_2_ and N_2_/H_2_ atmospheres induces comparable changes in the film composition. GI-XRD measurements show the (111), (200) and (220) reflections corresponding to the metallic Ir lattice for both PDA conditions at 500 °C, revealing that the amorphous IrO_*x*_ layer is transformed into metallic Ir. This result is corroborated by both XPS and AFM measurements. The O content in the films sharply decreases, with the majority of the O 1s spectrum being dominated by Si-bound-O signal originating from the native oxide of the substrate. AFM measurements reveal that the uniform films are transformed into islands by dewetting from the surface, as is characteristic for metallic thin films. This transformation is observed after PDA under N_2_/H_2_ at 300 and 500 °C as well as under N_2_ at 500 °C. These results are indicative of the reduction of the IrO_*x*_ and formation of metallic Ir, which already occurs at an annealing temperature of 300 °C. The atomic percentage of C increases slightly, due to the reduced percentage of O atoms, but the absolute number of C atoms incorporated in the film decreases. The N content is also significantly reduced, which is in line with expectations, as the N atoms in these films are primarily bound to O and C.

After PDA in O_2_ atmosphere at 500 °C, (110), (101), (200), and (211) reflections of rutile IrO_2_ are detected by GI-XRD, showing that the amorphous IrO_*x*_ film crystallizes during the PDA treatment. Shifts in the Ir 4f and O 1s XPS peaks toward lower binding energies reveal that the film converts from a compositional mix of primarily IrO_*x*_ with some IrO_2_, to a mix of primarily stoichiometric IrO_2_ with some IrO_*x*_.^44,45^ This is also reflected in the Ir-to-O ratio, which aligns with stoichiometric IrO_2_ after PDA at 500 °C. Furthermore, PDA helps to reduce the C-content from 5 at. % to 1 at. % and completely removes the N impurities from the films. AFM measurements reveal that the surface of the film remains virtually unaltered during PDA in O_2_ at 300 °C, but that the roughness increases at 500 °C, in line with expectations for the crystallization of an amorphous film.

Overall, PE-SALD of IrO_*x*_ and subsequent PDA in O_2_ atmosphere is a very promising strategy for preparing stable and pure crystalline IrO_2_ for OER. In the case of other applications where metallic Ir is preferred, annealing in N_2_ or N_2_/H_2_ atmosphere instead might be useful.

### Silicon vs Titanium Substrates

For use as a porous transport electrode (PTE), IrO_*x*_ is deposited directly onto the porous transport layer (PTL), which is typically made of a Ti felt or mesh. In this section, IrO_*x*_ films were deposited on Ti and Si substrates to compare the growth on these material surfaces and the resulting film compositions. Si wafers with 500 nm of sputtered Ti with native oxide, and standard Si wafers with native oxide were used for this. Because of the high surface roughness of these substrates, SE and XRR proved to be challenging to determine film thickness, so XRF was used to probe the Ir loading instead and served as a measure for the amount of deposited material.

Figure 9 shows 200 cycles of IrO_*x*_ deposited on Si and Ti substrates. AFM images (Figure 9a-d) show large differences in the starting surfaces, but the IrO_*x*_ deposition barely influences the surface roughness. The IrO_*x*_ deposition on Ti does have some smoothening effect, as can be seen in the decreased root-mean-square roughness *R*_q_, as expected for ALD.^59^ The roughness of the Si substrate is already below the detection limit of the AFM setup. XRF measurements (Figure 9e) show a slightly higher Ir loading on Ti than on Si. This difference is likely due to the difference in roughness of the starting surface, since a rougher surfaces with larger surface area allows for more precursor and co-reactant adsorption each ALD cycle. These results suggest that there is no large difference in nucleation delay on the two different substrates. XPS survey scans (Figure 9f) reveal that the deposited films have the same chemical composition, and high-resolution scans of the O 1s (Figure 9g) and Ir 4f (Figure 9h) peaks show that the oxidation states of these elements are identical. Overall, the PE-SALD IrO_*x*_ process shows the same behavior on Si and Ti substrates.

**Figure 9:**
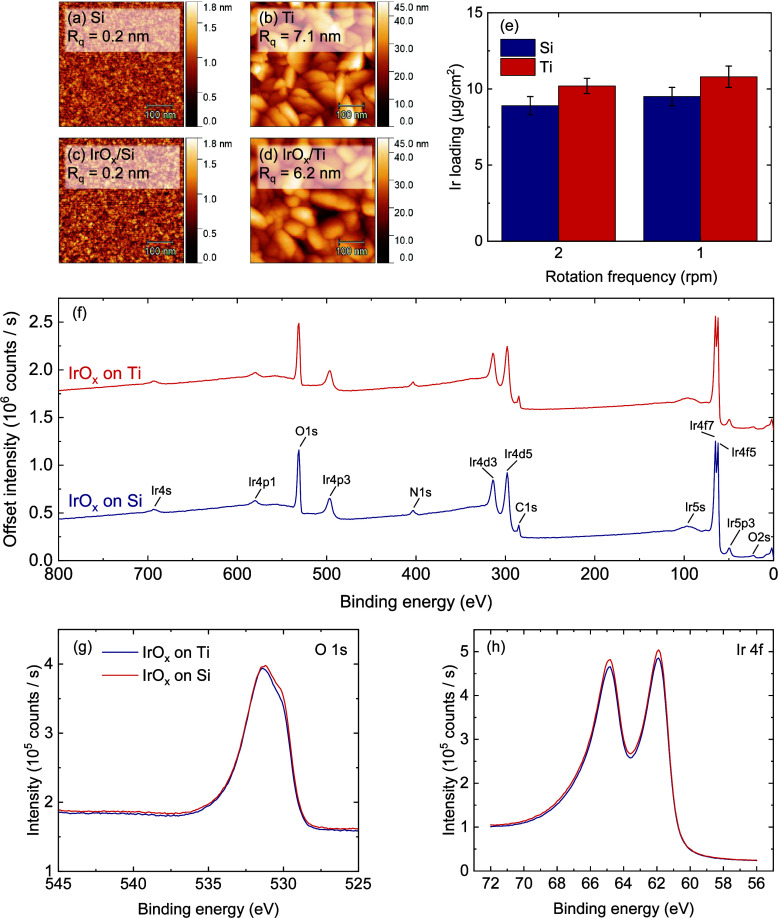
Comparison of 200 cycles PE-SALD IrO_*x*_ on Si and Ti substrates. AFM images of (a) pristine Si and (b) pristine Ti, and of (c) IrO_*x*_ on Si and (d) IrO_*x*_ on Ti. (e) Ir loading as determined by XRF. XPS surface scans: (f) survey spectrum, (g) high-resolution O 1s scan, (h) high-resolution Ir 4f scan. All data is for depositions with a precursor carrier-gas flow of 150 sccm and a rotation frequency of 2 rpm, except in (e), where also data from depositions with a precursor carrier-gas flow of 75 sccm and a rotation frequency of 1 rpm is included.

### Conformality

For the preparation of PTEs for application in PEM cells, it is essential that the ALD process is relatively conformal, i.e., the ability to deposit equal film thicknesses along the surfaces of 3D structures. The conformality of the IrO_*x*_ process was tested by depositing inside lateral high-aspect-ratio (LHAR) trench structures (Figure 10a) with a height of 500 nm. Four depositions were performed, each with a unique combination of either high or low precursor dose, and either high or low plasma dose (Table 1). Optical microscopy images were taken of the resulting films, on which gray scale analysis was performed to obtain thickness profiles. These thickness profiles (Figure 10b) show that the plasma exposure time largely determines the conformality, with 50%-thickness penetration depths (PD^50%^) of 6.3 and 7.8 μm for 3.9 s of plasma and 3.5 and 4.4 μm for 2.0 s of plasma. Additionally, the precursor exposure appears to play a minor role: lower exposure results in slightly higher penetration depth. An overview of these values and the corresponding achieved aspect ratios (AR) and equivalent aspect ratios (EAR) is shown in Table 1. The EAR is the equivalent aspect ratio that can be achieved with the same reactant dose in a cylindrical pore. For a wide trench $\mathrm{AR}=\frac{L}{h}$ and $\mathrm{EAR}=\frac{L}{2h}$ , where *L* is the trench length, and *h* is trench height.^60^ These results reveal that the limiting factor for the conformality of this process was the plasma step. Processes with such behavior are often called recombination limited, since a significant loss mechanism of the plasma radicals—besides the ALD surface reactions—is through recombination of the radicals upon collision with a surface. The oxygen radical recombination probability *r* is strongly dependent on the surface material (e.g., *r*_SiO_2__ ≈ *r*_TiO_2__ = ∼ 10^–5^ – 10^–4^, *r*_Al_2_O_3__ ≈ *r*_HfO_2__ = 10^–3^ – 10^–1^), which translates to large differences in conformality among various PE-ALD processes.^61–63^

**Table 1:** Description of Depositions in Lateral High-Aspect-Ratio (LHAR) Structures and Metrics for Their Conformality, i.e., 50%-Thickness Penetration Depth (PD^50%^), Coated Aspect Ratio (AR), and Coated Equivalent Aspect Ratio (EAR)^a^

Sample	Rotation frequency (rpm)	Flow through the precursor bubbler (sccm)	Precursor exposure (L)	Plasma exposure time (s)	PD^50%^ (μm)	Achieved AR	Achieved EAR
A	4	150	1.3 × 10^4^	2.0	4.4	8.8	4.4
B	4	300	2.6 × 10^4^	2.0	3.5	7.0	3.5
C	2	75	1.3 × 10^4^	3.9	7.8	15.6	7.8
D	2	150	2.6 × 10^4^	3.9	6.3	12.6	6.3

a Coated AR is defined as $\mathrm{AR}=\frac{{\mathrm{PD}}^{50\%}}{h}$ , where in this case *h* = 500 nm.

**Figure 10:**
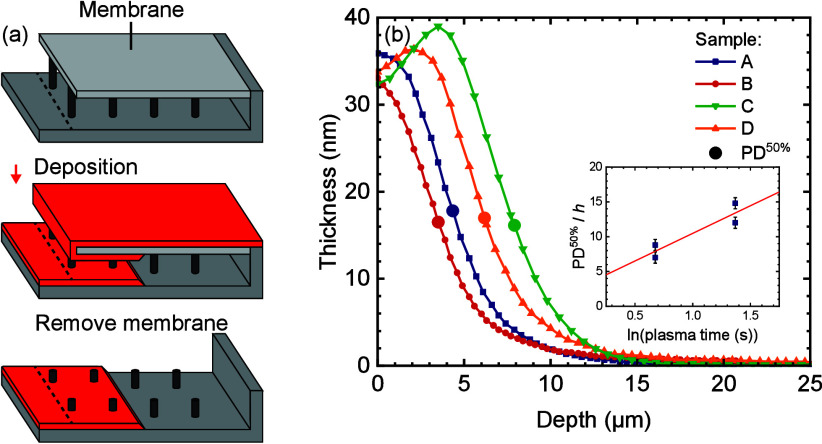
(a) Schematic of the PillarHall lateral high-aspect-ratio (LHAR) trench structure. The top wall is a membrane which can be removed after deposition to analyze the deposited film. (b) Measured thickness profiles of the deposited films as a function of depth inside the structure. Profiles were obtained through gray scale analysis of optical microscopy images and calibrated using SE measurements on planar substrates. The inset shows the 50%-thickness penetration depth (PD^50%^) scaled to the cavity height as a function of the natural logarithm of the plasma exposure time, used to estimate the recombination probability.^61^

The penetration depths obtained for various plasma exposure times can be used to determine *r* directly. For oxygen radicals from an atmospheric O_2_/N_2_ plasma inside a 500 nm high trench, as used in this study, this is done using

1 $$\frac{{\mathrm{PD}}^{50\%}}{h}\left(t\right)=\frac{1}{\sqrt{4.26r}}\ln\left(\frac{t}{t_{50\%}}\right)$$

where *h* is the trench height, *t* is the plasma exposure time, and *t*_50%_ is the plasma exposure time required for 50% saturation on a planar substrate.^63^ By plotting the measured PD^50%^/*h* as a function of the natural logarithm of the plasma exposure time, *r* is extracted from the slope (Figure 10b inset). From the presented IrO_*x*_ deposition data, *r* is estimated to be 3 × 10^–3^, with an uncertainty range of 2 × 10^–2^–1 × 10^–3^. While the uncertainty range is fairly large, due to the limited number of depositions with limited penetration depths, this method is still useful to determine the order of magnitude of *r* as this value can vary significantly between material systems. The moderate *r*-value reflects that the conformality of PE-ALD IrO_*x*_ is better than those of PE-ALD Al_2_O_3_ and HfO_2_, but not as good as those of PE-ALD TiO_2_ and SiO_2_.^61–63^ The use of atmospheric pressure PE-SALD can be beneficial for rapidly coating structures with moderate aspect ratios because of the high radical density in the atmospheric plasma.^63^ However, longer plasma exposure is expected to be required for higher aspect ratios due to slower diffusion at atmospheric pressure as compared to low pressure PE-ALD.

The target penetration depth in the PTL for application in PEM water electrolyzers is only a few μm, since the OER only takes place in close proximity of the catalyst, membrane, and PTL. Any deposition deeper inside the PTL leads to higher Ir-loading, while the protons generated deeper within the PTL are too far away from the membrane to be efficiently extracted from the half-cell. Although a direct comparison with the LHAR results is difficult since PTLs typically have a less well-defined geometry with pores of varying aspect ratios, the conformality of the process appears to be sufficient for this application. Since the conformality is limited by the plasma step, the Ir dose can be reduced to values close to the saturation value on planar substrates in order to increase precursor utilization efficiency and reduce costs.

## Conclusions

In this work, an SALD IrO_*x*_ process was developed using (EtCp)­Ir­(CHD) and O_2_/N_2_ plasma. The growth exhibits saturating behavior, with a GPC of 0.66 Å at 150 °C. Substantial amounts of N impurities, bound to O and C atoms, were detected in the films deposited at standard conditions, which are thought to originate from the O_2_/N_2_ plasma. The process shows a large deposition-temperature dependence between temperatures of 80 to 200 °C. With increasing temperatures, the C impurity level decreases, while the crystallinity increases. From deposition temperatures around 150–180 °C, metallic Ir clusters are formed, which increase in size with increasing temperature. Further reduction of impurities and increase of crystallinity is obtained through increasing the plasma exposure beyond the saturation value. This prolonged plasma results in a lower GPC of 0.57 Å, and a denser film with improved conductivity. The process exhibits moderate conformality compared to other PE-ALD processes. The recombination probability of oxygen radicals on the deposited IrO_*x*_ surface during the deposition is estimated to be 3 × 10^–3^. Comparing this value with values reported for other materials using the same method, the following trend is established: TiO_2_ < SiO_2_ < IrO_2_ < Al_2_O_3_ < HfO_2_.

A short PDA in O_2_ at 500 °C has been found to be an effective method for crystallizing the deposited IrO_*x*_, creating stoichiometric polycrystalline IrO_2_. Furthermore, the N impurities are completely removed from these films, while their C impurity content is significantly reduced. The process can be applied in the fabrication of PTEs for PEM water electrolysis as it is compatible with Ti substrates and can conformally coat around 10 μm inside LHAR test structures using moderate plasma exposure times. The actual penetration depth inside PTLs will be highly dependent on the shape of pores in the layer and their aspect ratio. Overall, the approach of preparing high-quality ultrathin IrO_2_ films through SALD of IrO_*x*_ and subsequent PDA in O_2_ is highly promising for producing PEM cells with reduced Ir loading. Future research should investigate the production of PTEs using this approach, their application in PEM cells, and the evaluation of their performance.

## Supplementary Material



## Notes

Additional text and figures (Figures S1–3) regarding the dielectric functions of the SE model used, precursor exposure and exposure time calculations, the linear growth of the process, and detailed XPS spectra for the plasma exposure time series (PDF)
