# Supplementary material for: Spatial Atomic Layer Deposition of IrO x Using (EtCp)Ir(CHD) and Atmospheric O2/N2 Plasma
Source: J Phys Chem C Nanomater Interfaces. 2025 Nov 27;129(49):21859–70. doi: 10.1021/acs.jpcc.5c06502 (PMC12706793; doi:10.1021/acs.jpcc.5c06502)
Supplement: Supplementary file 1 [file jp5c06502_si_001.pdf]

Spatial Atomic Layer Deposition of IrO_x Using (EtCp)Ir(CHD) and Atmospheric O_2/N_2 Plasma

Mike L. van de Poll[1], Jie Shen[2], Paul Poodt[1,3], Fieke van den Bruele[2], Wilhelmus M.M. Kessels[1], Bart Macco[1,*]

[1] *Department of Applied Physics and Science Education, Eindhoven University of Technology, 5600 MB Eindhoven, The Netherlands*

[2] *TNO/Holst Centre, High Tech Campus 31, 5656 AE Eindhoven, The Netherlands*

[3] *SparkNano B.V., Esp 266, 5633 AC Eindhoven, The Netherlands*

* *Email: b.macco@tue.nl*

Spectroscopic ellipsometry model

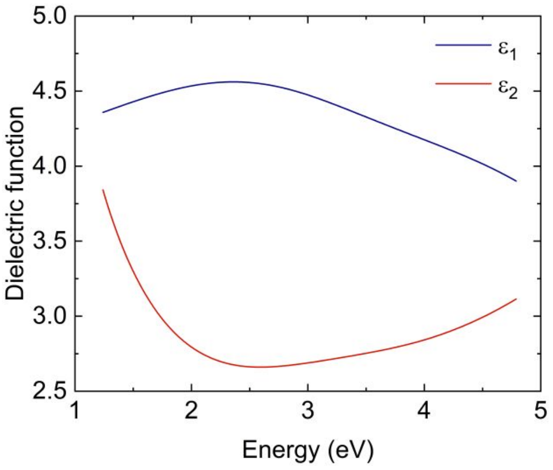

Figure S1: Dielectric functions obtained through modeling of the spectroscopic ellipsometry data for an IrO_x film deposited using a rotation frequency of 2 rpm and precursor-carrier flow of 150 sccm (2.6×10^4 L precursor exposure).

Precursor exposure and exposure time calculations

Precursor exposures E were calculated using $E = 10^{-6} p_{\mathrm{prec}} \times t$, where p_{prec} is the precursor partial pressure in Torr and t is the exposure time in s. The exposure is expressed in Langmuir (L). The precursor partial pressure is obtained from $p_{\mathrm{prec}} = p_0 \frac{Q_{\mathrm{prec}}}{Q_{\mathrm{in}}+Q_{\mathrm{prec}}}$, where p_o is atmospheric pressure, Q_{in} is the carrier-gas flow through the precursor bubbler, and Q_{prec} is the precursor flow calculated from $Q_{\mathrm{prec}} = Q_{\mathrm{in}} \frac{p_{\mathrm{vap}}}{p_0+p_{\mathrm{vap}}}$, where p_{vap} is the vapor pressure of the precursor.

With this calculation, a precursor pick-up efficiency of 1 is assumed. This assumption is made in most reports in the literature where flux or exposure is calculated. The pick-up efficiency in this work is enhanced by using a dip tube bubbler, rather than a flow-over vessel.

The exposure times are defined as the precursor/plasma slot widths divided by the substrate speed. The slots run from the exhaust slit on the left side of the precursor/plasma slit to the exhaust slit on the right side of the precursor/plasma slit. Because of the rotating-table design, the substrate speed and therefore exposure times are dependent on the position with respect to the center of the table. The

middle of each substrate was always placed at 40 mm from the center, and this distance was also used for all calculations. The slot widths are 12 mm and 32 mm for the precursor and plasma slots, respectively. As an example, at 5 rpm rotation frequency, this results in a precursor exposure time of 0.58 s and plasma exposure time of 1.6 s. Measurements were always taken from the center of the substrates to align with the calculated exposures at this position.

Linear growth

IrO_x films were deposited at 200 °C using a rotation frequency of 5 rpm and a varying number of cycles (Figure S2). The process exhibits linear growth. The data suggests a nucleation delay of around 50 cycles, which matches the nucleation delay observed by Di Palma *et al.* for the temporal ALD equivalent of this process.[1]

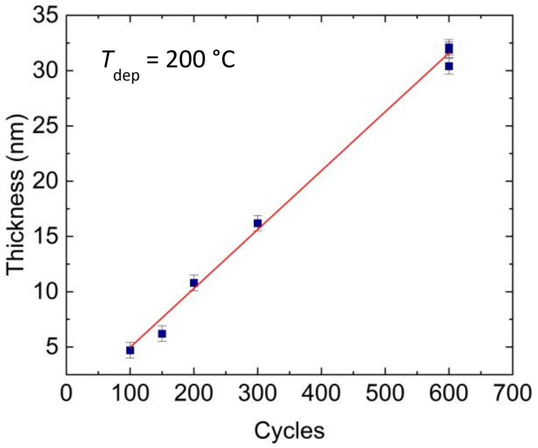

Figure S2: Thickness plot of PE-s-ALD IrO_x at 200 °C for a varying number of ALD cycles.

XPS spectra for plasma exposure time series

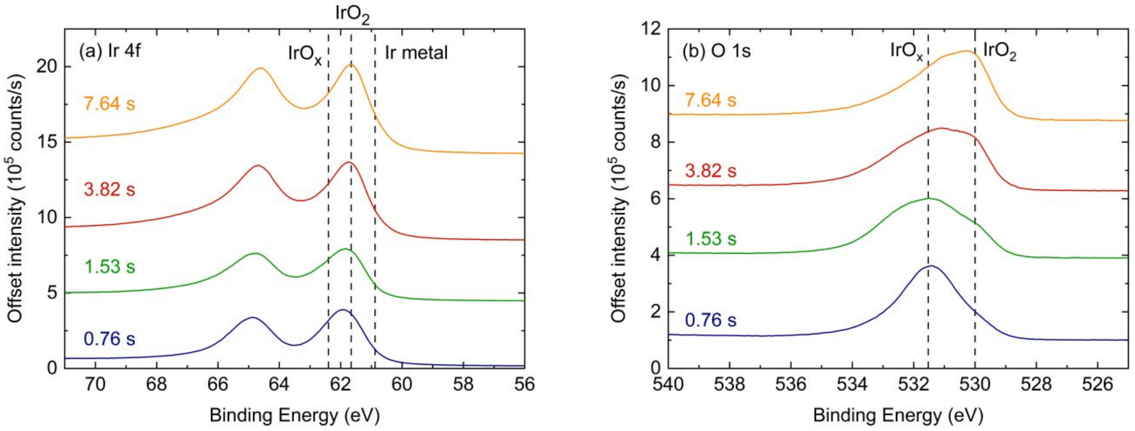

Figure S3: (a) Ir 4f and (b) O 1s XPS spectra for PE-s-ALD IrO_x using various plasma exposure times.

References

(1) Di Palma, V.; Pianalto, A.; Perego, M.; Tallarida, G.; Codegoni, D.; Fanciulli, M. Plasma-Assisted Atomic Layer Deposition of IrO2 for Neuroelectronics. *Nanomaterials* 2023, *13* (6), 976. https://doi.org/10.3390/NANO13060976/S1.
